# Oxidative balance score reflects vascular endothelial function of Chinese community dwellers

**DOI:** 10.3389/fphys.2023.1076327

**Published:** 2023-04-17

**Authors:** Jianhua Liu, Lingxiao He, Aozhe Wang, Yuanyuan Lv, Hui He, Chenghao Wang, Kaiyu Xiong, Li Zhao

**Affiliations:** ^1^ Department of Exercise Physiology, Beijing Sport University, Beijing, China; ^2^ College of Physical Education, WeiFang University, WeiFang, Shandong, China; ^3^ School of Public Health, Xiamen University, Xiamen, Fujian, China

**Keywords:** oxidative balance score, vascular endothelial function, lifestyle OBS, dietary OBS, Chinese community dwellers

## Abstract

**Background:** The oxidative balance score (OBS) is a composite estimate of the overall pro- and antioxidant risk status in an individual. The aim of this study is to explore the association between the OBS and vascular endothelial function in Chinese community dwellers.

**Methods:** In total, 339 community dwelling adults (aged 20–75 years) were recruited in this study. The overall OBS was calculated on the basis of 16 pro- and antioxidant factors related to diet (measured by fasting blood samples) and lifestyle (evaluated by questionnaires). The dietary OBS and lifestyle OBS were calculated on the basis of the corresponding components. Serum iso-prostaglandin F2α (FIP) was measured to evaluate the oxidative stress degree, and brachial artery blood flow-mediated dilation (FMD) was measured for vascular endothelial function. The FIP and FMD levels were dichotomized as “low” or “high” using the corresponding median values (low FIP, *n* = 159; high FIP, *n* = 180; low FMD, *n* = 192; and high FMD, *n* = 147). The components of the OBS were compared between the stratified FIP and FMD groups. Logistic regression was used to analyze the OBS associations with FIP and FMD.

**Results:** The higher overall OBS and dietary OBS were associated with lower FIP (*p* < 0.001), whereas the higher overall OBS (*p* < 0.01) and dietary OBS (*p* < 0.05) were associated with higher FMD. The lifestyle OBS was not associated with FIP and FMD (*p* > 0.05). Except for the body mass index (BMI) and low physical activity, all other OBS components were significantly different between the low FIP and high FIP groups (*p* < 0.05). Four diet-related antioxidants (α-carotene, zeaxanthin, α-tocopherol, and γ-tocopherol) showed significant differences between the high and low FMD groups (*p* < 0.05).

**Conclusion:** The decreasing OBS level was associated with low endothelial function and high oxidative stress. The dietary OBS, rather than the lifestyle OBS, was more closely associated with endothelial function.

## 1 Introduction

Oxidative stress (OS) is a condition that occurs when there is an imbalance between the production of free radicals and defense of antioxidants, resulting in excessive accumulation of reactive oxygen species (ROS) and reactive nitrogen species (RNS) (Niki, 2018). It is reported that OS plays an important role in initiating and mediating chronic non-communicable pathophysiology diseases, such as cardiovascular disease (CVD), hypertension, and type 2 diabetes (Stanner et al., 2004). The presence of OS can alter protein structure and hinder enzymes such as the endothelial nitric oxide synthase (eNOs), thereby resulting in dysfunction and inability of the endothelium to release and respond to vasodilators (Cathcart, 2004; Yokoyama, 2004; Antelava et al., 2005). Although the pathophysiology of CVD is extremely complex and multifactorial, local injury to, or dysfunction of, endothelial cells appear to be an important factor in the initiation of atherosclerosis (Dokken, 2008). Besides, the mitochondrial respiratory chain, NADPH oxidase, an uncoupled endothelial nitric oxide (NO) synthase, and xanthine oxidase also produce ROS that causes elevated levels of OS in the vascular system of people with high risk factors (Griendling et al., 2021). An increased formation of ROS from all layers of the vascular wall may alter the vascular function and contribute to the development and progression of CVD (Cersosimo and DeFronzo, 2006). OS may be the earliest biological event in chronic CVD pathological cascade and is responsible for triggering all other pathologies, indicating that reducing OS may be a viable approach in disease treatment.

OS process is affected by environment, lifestyle, and diet. Cigarettes, ionizing radiation, environment pollution, medicine, iron, saturated fatty acid, and other prooxidants all boost the OS process. Fitting exercise and antioxidants, which include vitamin C, vitamin E, vitamin A, carotenoids, and selenium, all reduce the OS degree (Valko et al., 2007). Notably, although lab work has demonstrated that antioxidants can slowdown the progression of hypertension, further clinical evidence is still required (Pietta, 2000; Padayatty et al., 2003; Stahl and Sies, 2003), and clinical trials have failed to find any benefit of antioxidant supplements in hypertension treatment (Heart Protection Study Collaborative Group, 2002; Rodrigo et al., 2013) or cardiovascular disease prevention (Yusuf et al., 2000; Cortés-Jofré et al., 2020). Such an inconsistency between lab and clinical research implies that the impact of the body’s oxidative status on the cardiovascular system is complex and not exclusively determined by antioxidant factors. In fact, the body’s oxidative status is the result of interactions among multiple pro-/antioxidant factors. Therefore, studies on OS-related pathophysiology should combine information of both pro- and antioxidant factors, rather than a single factor. The oxidative balance score (OBS) is an approach that evaluates the overall oxidative status in the body *via* the combined status of pro- and antioxidants (Van Hoydonck et al., 2002). Previous studies have used 12–14 pro- and antioxidant factors related to nutrients, lifestyle, body composition, and medical history for OBS calculation, with antioxidants contributing positively to the value and prooxidants contributing negatively to the value (Kong et al., 2015; Noruzi et al., 2021). The higher OBS has been associated with a lower risk of various non-communicable diseases and adverse outcomes, such as hypertension (Annor et al., 2015), colorectal adenoma (Dash et al., 2013), prostate cancer (Lakkur et al., 2014), chronic kidney disease, and all-cause mortality (Ilori et al., 2015; Kong et al., 2015). In the development of these disease conditions, OS-induced endothelial dysfunction, which is usually estimated by flow-mediated dilation (FMD) of the brachial artery (Thijssen et al., 2019; Lv et al., 2020), is one of the earliest signs. Such endothelial dysfunction might be affected by serum 8-iso-prostaglandin F2α (FIP), a specific sensitive indicator of OS that is involved in the pathophysiological changes of atherosclerosis (Mueller et al., 2004; Lepara et al., 2020). FMD of the brachial artery is a widely used parameter in evaluating the vascular endothelial function (Thijssen et al., 2011) and independently predicting the risk of cardiovascular diseases (Donato et al., 2018). Notably, although pro- and antioxidant factors are associated with endothelial dysfunction risk, the relationship between the holistic effect of these factors (evaluated by the OBS) and vascular endothelial function has not been reported. Additionally, associations between the OBS subgroups (such as the lifestyle- and diet-related OBS) and vascular endothelial functions are also informative in understanding how our living habits are associated with vascular endothelial conditions from the OS aspect.

Therefore, the present study calculated the OBS based on lifestyle- and diet-related pro- and antioxidant factors to investigate the OBS association with vascular endothelial function. Moreover, associations between the OBS components and endothelial function were analyzed to identify potential factors for vascular endothelial dysfunction prevention.

## 2 Materials and methods

### 2.1 Study population

A total of 382 residents from the Beijing Shunyi District volunteered to participate in this study. Of which, 42 people were excluded from the final analysis, in them 19 did not provide a blood sample, 16 did not complete the FMD measurement, and 7 did not complete the questionnaire. Finally, 339 Chinese Han ethnicity participants who could complete all the tests and questionnaires were included in this study, of whom 142 were male and 197 were female. Participants were recruited from a community in Beijing on a voluntary basis. The inclusion criteria were 1) aged 20–75 years, 2) long-term residents of the area, and 3) could complete all the test procedures and questionnaires as required. There were no special requirements for the health or disease status of the participants. The study protocol was approved by the Ethics Committee of Beijing Sport University (2017001H), and the study procedures were carried out in accordance with the Declaration of Helsinki. All participants were fully informed about the study and written consent forms were obtained from them.

### 2.2 Questionnaires

Some of the OS factors were collected by questionnaires. The questionnaires consisted of the long-form International Physical Activity Questionnaire (IPAQ) (Chinese version), which has been proven to have good validity and reliability in the Chinese population (Qu and Ji, 2004), and other questionnaires regarding the demographic characteristics (age, sex, and education), medical history (normal chronic diseases and use of medications, especially cardiovascular and cerebrovascular diseases), medication history (aspirin and NSAIDs used), smoking and alcohol consumption, family medical history, physical activity level, diet, and social habits. The questionnaires were completed by in-person interviews.

### 2.3 Basic medical checkup

Basic medical checkup included tests for height, weight, resting heart rate, and systolic (SBP) and diastolic (DBP) blood pressures. All instruments were calibrated before testing.

### 2.4 Blood tests and laboratory analyses

All subjects were required to fast at least 12 h beforehand. Three milliliters of venous blood was drawn into a red-top tube. The blood sample was rested at room temperature for 10 min and then centrifuged for 10 min at 3000 r/min speed. The serum was collected, aliquoted, and stored at −80°C for later analysis.

Triglyceride (TG), total cholesterol (TC), low-density lipoprotein (LDL), high-density lipoprotein (HDL), and blood glucose (BG) were measured by routine blood test. FIP, ferritin (FER), Vit C, unsaturated fatty acid-3 (ω-3), and unsaturated fatty acid-6 (ω-6) were measured with the double-antibody one-step sandwich method enzyme-linked immunosorbent assay (ELISA) using Beckman AU5800 (Beckman Coulter, California, America). Serum α-carotene, β-carotene, β-cryptoxanthin, zeaxanthin, α-tocopherol, and γ-Toc were measured by high-performance liquid chromatography tandem mass spectrum using Shimadzu LC-20AD (Shimadzu, Kyoto, Japan) and AB Sciex API 3200MD Trap (Sciex, Boston, America).

### 2.5 Oxidative balance score

The overall OBS of this study was calculated by 16 pro- and antioxidant factors. In particular, there are two dietary prooxidants: ω-6 and FER(Toborek et al., 1996; Ehara et al., 2001; Ghosh et al., 2006; Van Beelen et al., 2006; Syrovatka et al., 2011); eight dietary antioxidants: α-carotene, β-carotene, β-cryptoxanthin, zeaxanthin, α-tocopherol, γ-tocopherol, Vit C, and ω-3 (Rimm et al., 1993; Frei, 1994; Upritchard et al., 2003; Kinlay et al., 2004; Tamimi et al., 2005; Cai et al., 2010; Milani et al., 2017; Elvira-Torales et al., 2019; Snezhkina et al., 2019; Stupin et al., 2019; Yimcharoen et al., 2019; Drenjančević and Pitha, 2022; Khutami et al., 2022); three lifestyle prooxidants: BMI, smoking history, and alcohol consumption (Keaney et al., 2003; Furukawa et al., 2004; van der Vaart et al., 2004; Frohnert et al., 2011; Caliri et al., 2021); and three lifestyle antioxidants: physical activity level, aspirin, and NSAID use frequency (Kaur and Geetha, 2006; Radak et al., 2008). Factors with continuous variables were divided into categories based on the tertile values. Antioxidant factors in the lower, middle, and upper tertile groups were assigned zero, one, and two points, respectively. Reversely, prooxidant factors in the lower, middle, and upper tertile groups were assigned two, one, and zero points, respectively. The scores of the factors with categorical variables were assigned 0 to −2 according to specific conditions. The detailed scoring criteria are shown in Supplementary Table S1. The dietary OBS (ranging from 0 to 10) was calculated by summing the scores of the dietary pro- and antioxidant factors, and the lifestyle OBS (ranging from 0 to 6) was calculated by summing the scores of the lifestyle pro- and antioxidant factors.

### 2.6 Tests of vascular endothelial function

Brachial artery blood FMD was measured to estimate the vascular endothelia function. In particular, pressure was applied to the forearm to increase blood flow shear stress, resulting in increased synthesis and release of NO that causes vasodilation. Ultrasound (UNEX EF, Nagoya, Japan) was used to record the inner diameter changes of the blood vessels during the process, and the percentage change of the inner diameter was calculated as FMD. FMD lower than the threshold value (i.e., 5%) of the ultrasound machine indicated hypofunction of endothelium cells. The measurement was performed by a specialized technician.

### 2.7 Covariates

Age, sex, education level, TG, TC, LDL, HDL, SBP, DBP, cardiovascular diseases (yes/no), and diabetes (yes/no) were controlled in the analysis.

### 2.8 Statistical analysis

Data are presented as mean ± standard deviation. Group comparisons were performed using the *t*-test, chi-square test, or one-way analysis of variance (ANOVA) as appropriate. FIP and FMD were dichotomized into the “low” and “high” groups using their respective median values as the cutoff. The OBS was analyzed as overall OBS, dietary OBS, and lifestyle OBS tertiles. Logistic regression was used to analyze the OBS associations with FIP and FMD with adjustments made for covariates. Sensitivity analysis was further performed to complete each OBS component level between the low and high FIP and FMD groups (dichotomized by the corresponding median values) by independent samples *t*-test. Statistical significance was determined at a two-sided *p*-value of 0.05. Statistical analysis was performed using the SPSS statistical software version 23.0.

## 3 Results

### 3.1 Characteristics of participants

The demographic, dietary, and lifestyle characteristics of the participants are shown in Table 1. There are more female participants (58.11%), and their heights and weights were significantly lower than those of the male participants. There were no significant differences in BMI, Vit C, ω-3, ω-6, β-cryptoxanthin, zeaxanthin, α-tocopherol, γ-Toc, and FIP between males and females. Higher β-carotene and lower α-carotene and FER were found in males than in females. Higher proportions of smoking and alcohol overconsumption were found in males than in females. The overall physical activity in male participants was lower than that in female participants. The proportion of females engaged in high physical activity was higher than that of males. There were no significant differences in the use of aspirin and NSAID between males and females.

**TABLE 1:** Descriptive characteristics of participants.

	All adults (*n* = 339)	Male (*n* = 142)	Female (*n* = 197)
Age (years)	53.0 ± 13.1	51.8 ± 14.6	53.9 ± 12.3
Height (cm)	160.05 ± 8.37	166.90 ± 7.52	155.12 ± 5.45**
Weight (kg)	67.91 ± 11.02	72.48 ± 10.71	64.61 ± 10.22**
Body mass index (kg/m^2^)	26.55 ± 3.79	26.12 ± 3.52	26.85 ± 3.92
Vitamin C (umol/L)	31.71 ± 9.26	31.63 ± 9.77	31.75 ± 9.02
ω-3 Fatty acid (umol/L)	80.03 ± 21.37	81.19 ± 25.29	79.44 ± 19.12
ω-6 Fatty acid (umol/L)	12.84 ± 3.42	12.52 ± 3.58	13.01 ± 3.34
α-Carotene (ng/mL)	19.71 ± 4.77	18.68 ± 5.52	20.23 ± 4.26*
β-Carotene (ng/mL)	41.35 ± 10.89	44.47 ± 12.23	39.75 ± 9.81**
β-Cryptoxanthin (ng/mL)	24.68 ± 7.93	24.63 ± 8.55	24.70 ± 7.62
Zeaxanthin (ng/mL)	29.53 ± 8.39	28.35 ± 8.43	30.14 ± 8.33
α-Tocopherol (ng/mL)	1795.85 ± 334.89	1767.52 ± 415.97	1810.37 ± 284.98
γ-Tocopherol (ng/mL)	1068.37 ± 257.13	1082.92 ± 285.36	1060.91 ± 241.99
Ferritin (ng/mL)	57.38 ± 8.56	55.39 ± 8.84	58.39 ± 8.26**
8-Iso-PGF_2α_ (pg/mL)	517.97 ± 78.41	523.27 ± 83.12	515.26 ± 76.01
Current smoking (%)	88 (25.96)	83 (58.45)	5 (2.54)**
Current alcohol user (%)	52 (15.34)	48 (33.80)	4 (2.03)**
Low physical activity (%)	24 (7.08)	17 (11.97)	7 (3.55)**
Medium physical activity (%)	101 (29.79)	47 (33.10)	54 (27.41)
High physical activity (%)	214 (63.13)	78 (54.93)	136 (69.04)**
NSAID user (%)	117 (34.51)	47 (33.10)	70 (35.53)
Aspirin user (%)	85 (25.07)	40 (28.17)	45 (22.84)

*p < 0.05, **p < 0.01, when compared with males based on the *t*-test for continuous variables and chi-square test for categorical variables.

### 3.2 Comparisons of OBS components in stratified FIP and FMD groups

Comparisons of all OBS components between the stratified FIP groups are shown in Table 2. All the eight dietary antioxidants were higher in the low FIP group than high FIP group. Both dietary prooxidants (i.e., ω-6 and FER) were lower in the low FIP group. The proportion of participants with lifestyle antioxidants (aspirin use frequency) and prooxidants (i.e., smoking and alcohol consumption) were lower in the low FIP group than in the high FIP group.

**TABLE 2:** Comparisons of OBS components between high and low FIP levels.

OBS components		Low FIP (*n* = 159)	High FIP (*n* = 180)	*p*-value
Prooxidant factors	BMI (kg/m^2^)	26.08 ± 4.29	26.98 ± 3.21	0.062
	Low physical activity (%)	13 (8.18)	11 (6.11)	0.459
	Smoking (%)	32 (20.12)	56 (31.11)	0.021
	Above-median alcohol user (%)	14 (8.81)	38 (21.11)	0.002
	Serum ferritin (ng/mL)	52.82 ± 8.80	61.01 ± 6.01	0.001
	ω-6 Fatty acid (umol/L)	11.79 ± 3.39	13.63 ± 3.16	0.001
Antioxidant factors	Vitamin C (umol/L)	35.13 ± 9.17	28.91 ± 8.39	0.001
	ω-3 Fatty acid (umol/L)	87.98 ± 22.49	73.60 ± 17.87	0.001
	α-Carotene (ng/mL)	21.14 ± 4.99	18.55 ± 4.26	0.001
	β-Carotene (ng/mL)	45.32 ± 10.50	38.29 ± 9.97	0.001
	β-cryptoxanthin (ng/mL)	26.69 ± 8.00	23.19 ± 7.48	0.001
	Zeaxanthin (ng/mL)	32.32 ± 8.47	27.30 ± 7.62	0.001
	α-Tocopherol (ng/mL)	1947.94 ± 353.58	1669.05 ± 257.58	0.001
	γ-Tocopherol (ng/mL)	1205.31 ± 257.67	958.08 ± 190.55	0.001
	Aspirin user (%)	21 (13.21)	64 (35.56)	0.001
	NSAID user (%)	51 (32.07)	66 (36.67)	0.375

Comparisons of all OBS components between the stratified FMD groups are shown in Table 3. Four dietary antioxidants (α-carotene, zeaxanthin, α-tocopherol, and γ-Toc) were higher in the high FMD group than the low FMD group, while other pro- and antioxidants failed to show differences between the FMD groups.

**TABLE 3:** Comparisons of OBS components between high and low FMD levels.

OBS components		Low FMD (*n* = 192)	High FMD (*n* = 147)	*p*-value
Prooxidant factors	BMI (kg/m^2^)	26.43 ± 3.70	26.83 ± 3.99	0.420
	Low physical activity (%)	15 (7.90)	9 (6.90)	0.490
	Smoking (%)	56 (29.60)	32 (21.30)	0.084
	Above-median alcohol user (%)	33 (17.50)	19 (12.70)	0.224
	Serum ferritin (ng/mL)	58.06 ± 7.86	56.09 ± 9.13	0.072
	ω-6 Fatty acid (umol/L)	12.60 ± 3.39	12.98 ± 3.39	0.389
Antioxidant factors	Vitamin C (umol/L)	30.83 ± 9.49	33.05 ± 8.90	0.064
	ω-3 Fatty acid (umol/L)	78.90 ± 21.96	82.10 ± 20.55	0.246
	α-Carotene (ng/mL)	19.12 ± 4.74	20.58 ± 4.74	0.018
	β-Carotene (ng/mL)	40.93 ± 10.57	42.40 ± 11.04	0.289
	β-Cryptoxanthin (ng/mL)	24.79 ± 8.30	24.86 ± 7.40	0.946
	Zeaxanthin (ng/mL)	27.97 ± 8.01	31.75 ± 8.43	0.001
	α-Tocopherol (ng/mL)	1754.24 ± 366.24	1856.16 ± 284.77	0.018
	γ-Tocopherol (ng/mL)	1043.20 ± 237.40	1111.85 ± 273.55	0.037
	Aspirin user (%)	55 (28.65)	30 (20.41)	0.083
	NSAID user (%)	69 (35.94)	48 (32.65)	0.528

### 3.3 Associations between different types of OBS, FIP, and FMD

The overall OBS was stratified by the upper tertile cutoff value of 19 and lower tertile cutoff value of 15 (Noruzi et al., 2021). The dietary OBS was stratified by the upper and lower tertile cutoff values of 13 and 8, respectively. The lifestyle OBS was stratified by the upper and lower tertile cutoff values of 8 and 6, respectively. Logistic regression on the stratified FIP groups (stratified by the median value of 516.73 pg/mL) showed that higher overall and dietary OBSs were associated with a lower chance of developing high FIP (Table 4). Particularly, the odds ratio of becoming the high FIP group was 0.17 (95% CI: 0.086–0.355) between the middle and lower overall OBS groups, and 0.054 (95% CI: 0.025–0.115) between the higher and lower overall OBS groups. The odds ratio of becoming the FIP group was 0.24 (95% CI: 0.123–0.467) between the middle and lower dietary OBS groups, and 0.041 (95% CI: 0.017–0.099) between the higher and lower dietary OBS groups. Similarly, logistic regression on the stratified FMD groups (stratified by the median value of 10.70%) showed that higher overall and dietary OBSs were associated with a lower chance of developing low FMD (Table 5). The odds ratio of becoming the low FMD group was 0.535 (95% CI: 0.289–0.992) between the middle and lower Overall OBS groups, and 0.397 (95% CI: 0.206–0.766) between the higher and lower overall BOS groups. The odds ratio of becoming the low FMD group was 0.455 (95% CI: 0.248–0.836) between the higher and lower dietary OBS groups.

**TABLE 4:** Results of logistic regression between OBS and FIP.

		AOR	95% CI	*p*-value
Overall OBS	Tertile 1	Ref	—	—
	Tertile 2	0.170	0.086–0.335	0.001
	Tertile 3	0.054	0.025–0.115	0.001
Dietary OBS	Tertile 1	Ref	—	—
	Tertile 2	0.240	0.123–0.467	0.001
	Tertile 3	0.041	0.017–0.099	0.001
Lifestyle OBS	Tertile 1	Ref	—	—
	Tertile 2	0.984	0.522–1.853	0.959
	Tertile 3	0.634	0.309–1.300	0.214

CI, confidence interval; AOR, adjusted odds ratio controlling for age, sex, education, fitness, tri-glycerides, total cholesterol, LDL-cholesterol, HDL-cholesterol, systolic pressure, diastolic pressure, cardiovascular disease, and diabetes. In addition, the dietary OBS was adjusted for lifestyle OBS components, and the lifestyle OBS was adjusted for dietary OBS components.

**TABLE 5:** Results of logistic regression between OBS and FMD.

		AOR	95% CI	*p*-value
Overall OBS	Tertile 1	Ref	—	—
	Tertile 2	0.535	0.289–0.992	0.047
	Tertile 3	0.397	0.206–0.766	0.006
Dietary OBS	Tertile 1	Ref	—	—
	Tertile 2	0.621	0.332–1.160	0.135
	Tertile 3	0.455	0.248–0.836	0.011
Lifestyle OBS	Tertile 1	Ref	—	—
	Tertile 2	0.571	0.312–1.044	0.069
	Tertile 3	1.061	0.513–2.196	0.873

CI, confidence interval; AOR, adjusted odds ratio, controlling for age, sex, education, fitness, tri-glycerides, total cholesterol, LDL-cholesterol, HDL-cholesterol, systolic pressure, diastolic pressure, cardiovascular disease, and diabetes. In addition, the dietary OBS was adjusted for lifestyle OBS components, and the lifestyle OBS was adjusted for dietary OBS components.

## 4 Discussion

The current study calculated the OBS based on 16 pro- and antioxidant factors to evaluate the influence of the ROS on the oxidative status in Chinese community dwellers. FIP was measured to estimate the OS level, and FMD was measured to evaluate the endothelial function. Our analyses showed that lower overall OBS and dietary OBS were associated with higher OS levels (i.e., a higher FIP value) and worse endothelial functions (i.e., a lower FMD value). Analyses on the OBS components showed that dietary and lifestyle pro- and antioxidant factors were associated with OS. Meanwhile, four dietary antioxidants (α-carotene, zeaxanthin, α-tocopherol, and γ-Toc) were associated with the endothelial function.

Recent studies have indicated that endothelial dysfunction may predict long-term atherosclerotic disease progression and cardiovascular event occurrence (El Assar et al., 2013). Although the mechanisms underlying endothelial dysfunction are multifactorial, cumulative evidence suggest that OS can directly affect the vascular function and tone by oxidative modification of proteins or nucleic acids. A major mechanism for the impact of OS on vascular tone is the decrease of NO bioavailability and/or signaling, leading to endothelial dysfunction. Additionally, excessive ROS may also promote vascular cell proliferation and migration, inflammation, and apoptosis, as well as extracellular matrix alterations (Schulz et al., 2011). Therefore, OS is a major cause of endothelial dysfunction (Incalza et al., 2018). The OBS has become a popular tool in evaluating the global OS status with diseases. Lower OBSs are associated with higher OS status and increased risk of hypertension (Annor et al., 2015), prostate cancer, and colon cancer (Kong et al., 2014). The current study extended the association between the OBS and OS by showing that the OBS was reversely associated with the OS status (reflected by serum FIP) in Chinese community dwellers. FIP is commonly used to evaluate lipid oxidative damage (Gào et al., 2018). FIP is a member of the F2-isoprostane family, which includes prostaglandin-like compounds produced by non-enzymatic peroxidation of arachidonic acid (Nourooz-Zadeh, 2008). Multiple studies have shown that FIP is a reliable marker of oxidative stress *in vivo* (Morrow, 2005; Milne et al., 2007). Considering the convenience of collecting data for the OBS, this parameter might be a potential surrogate of FIP for evaluating the OS status.

The OBS components are mainly derived from diet and lifestyle, where the former are nutrients while the latter are lifestyle-related factors, such as smoking, alcohol consumption, physical activity, and the use of medicine. To explore the impact of diet and lifestyle on endothelial function, the corresponding OBS was analyzed separately. The results have shown that the dietary OBS, rather than the lifestyle OBS, is significantly associated with endothelial function, indicating that nutrients might play a more important role than lifestyle in regulating endothelial function. Notably, four dietary antioxidants (α-carotene, zeaxanthin, α-tocopherol, and γ-Toc) have shown significant differences between high and low endothelial function groups. Such results are consistent with previous research on the supplementation of antioxidants and diseases (Ehara et al., 2001; Van Beelen et al., 2006) in which some pro- or antioxidants alone have no direct relationship with the occurrence and development of diseases. Meanwhile, a previous study found that the lifestyle OBS was more significantly associated with adenoma incidence than the dietary OBS, and the physical activity level in lifestyle was more important for colorectal adenoma than components in dietary (Kong et al., 2014). Such inconsistent findings of the dietary OBS and lifestyle OBS in previous disease studies have indicated that the importance of diet- and lifestyle-related OS might vary with diseases.

In this cross-sectional study, we examined the associations between FIP and the different types of OBSs and found that the dietary OBS had a strong, statistically significant inverse association with FIP, but the lifestyle OBS had no such association with FIP. In the analysis of the associations between the FIP and OBS components, we observed that all components showed significant differences between low FIP and high FIP groups except BMI and low physical activity. Such results are within our expectation since antioxidants can lower the body’s oxidative status and prooxidants exert the opposite effect. We also found a discrepant result that the proportions of aspirin use in the high FIP group were significantly higher than those in the low FIP group despite that the use of these medicines should have been in favor of lowering FIP. A possible explanation might be the bias of medicinal usage among participants. Specifically, people begin taking aspirin only after the onset of hypertension, hyperlipidemia, and other cardiovascular and cerebrovascular diseases that are closely related to the oxidative status. Consequently, people with comparatively high oxidative status tend to use aspirin, while others with a lower oxidative status do not take these medicines actively.

One of the strengths of this study is that the micronutrient levels were measured from samples instead of being estimated by food frequency questionnaire. Therefore, our OBS calculation on diet-related pro- and antioxidants is quite accurate. Separate analyses of the dietary OBS and lifestyle OBS is another novelty, which helped clarify the aspect of OBS that contributes more to endothelial function. Furthermore, the analyses of their associations with OS and vascular endothelial function helped explore the main factors influencing the vascular endothelial function to identify and propose preventive measures. The limitation of this study is that it is a cross-sectional and of limited sample size. Therefore, the current research conclusion should be treated with caution. In addition, the study population is limited to Chinese community dwellers, and some aspects of their lifestyle are less diverse, therefore it is unclear if these findings can be generalized to other populations. The OBS was limited to dietary/lifestyle exposures and failed to include endogenous enzymatic mechanisms (e.g., superoxide dismutase, glutathione peroxidase, and catalase), which might also affect the oxidative balance by clearing free radicals and reducing OS (Valko et al., 2016; Kattoor et al., 2017).

In conclusion, this study analyzed the association between the oxidative balance score and vascular endothelial function. The overall oxidative balance score reflects the body’s oxidative stress status and was found negatively associated with endothelial function. The dietary-related oxidative balance score was more closely associated with vascular endothelial function when compared with the lifestyle-related one.

## Data Availability

The raw data supporting the conclusion of this article will be made available by the authors, without undue reservation.
