# Supplementary material for: Oxidative balance score reflects vascular endothelial function of Chinese community dwellers
Source: Front Physiol. 2023 Apr 17;14:1076327. doi: 10.3389/fphys.2023.1076327 (PMC10150015; doi:10.3389/fphys.2023.1076327)
Supplement: Supplementary file 1 [file Table1.DOCX]

Supplementary Material

Table S1. OBS assignment scheme

| OBS Components | Assignment Scheme | | |
| --- | --- | --- | --- |
| Plasma α-carotene | 0=low (1^st^ tertile) | 1=medium (2^nd^ tertile) | 2=high (3^rd^ tertile) |
| Plasma β-carotene | 0=low (1^st^ tertile) | 1=medium (2^nd^ tertile) | 2=high (3^rd^ tertile) |
| Plasma β-cryptoxanthin | 0=low (1^st^ tertile) | 1=medium (2^nd^ tertile) | 2=high (3^rd^ tertile) |
| Plasma zeaxanthin | 0= low (1^st^ tertile) | 1=medium (2^nd^ tertile) | 2=high (3^rd^ tertile) |
| Plasma α-tocopherol | 0=low (1^st^ tertile) | 1=medium (2^nd^ tertile) | 2=high (3^rd^ tertile) |
| Plasma γ-tocopherol | 0=low (1^st^ tertile) | 1=medium (2^nd^ tertile) | 2=high (3^rd^ tertile) |
| Plasma Vitamin C | 0=low (1^st^ tertile) | 1=medium (2^nd^ tertile) | 2=high (3^rd^ tertile) |
| Plasma ω-3 fatty acid | 0=low (1^st^ tertile) | 1=medium (2^nd^ tertile) | 2=high (3^rd^ tertile) |
| Plasma ω-6 fatty acid | 2=low (1^st^ tertile) | 1=medium (2^nd^ tertile) | 0=high (3^rd^ tertile) |
| Serum ferritin | 2=low (1^st^ tertile) | 1=medium (2^nd^ tertile) | 0=high (3^rd^ tertile) |
| Body mass index | 0=obese | 1=overweight | 2=normal weight |
| Physical activity | 0=low (1^st^ tertile) | 1=medium (2^nd^ tertile) | 2=high (3^rd^ tertile) |
| Smoking | 0=current smoker | 1=former smoker | 2=never smoked |
| Alcohol use | 0=above median | 1=below median | 2=never drinked |
| Aspirin use | 0=no regular user | 1=unknown | 2=regular user |
| NSAID use | 0=no regular user | 1=unknown | 2=regular user |
